# The Impact of Immune-Modifying Treatments for Skin Diseases on the Immune Response to COVID-19 Vaccines: a Narrative Review

**DOI:** 10.1007/s13671-022-00376-3

**Published:** 2022-10-25

**Authors:** Su-Yi Liew, Timothy Tree, Catherine H. Smith, Satveer K. Mahil

**Affiliations:** 1 Guy’s and St Thomas’ NHS Foundation Trust, London, UK; 2 Faculty of Life Sciences and Medicine, Department of Immunobiology, King’s College London, London, UK; 3 St John’s Institute of Dermatology, Guy’s and St Thomas’ NHS Foundation Trust and King’s College London, London, UK

**Keywords:** COVID-19 vaccine, Immune-modifying therapy, Immunosuppression, Skin disease

## Abstract

***Purpose of Review*:**

SARS-CoV-2 has had a devastating global effect, with vaccinations being paramount in the public health strategy against COVID-19. Vaccinations have uncoupled infection from adverse COVID-19 outcomes worldwide. While immune-modifying therapies are effective for the management of skin diseases such as psoriasis and atopic dermatitis, these medications also impair protective immune responses. There has been longstanding uncertainty and concern over the impact of immune-modifying therapies on the effectiveness of vaccines; for example, it is well recognised that methotrexate impairs humoral responses to both influenza and pneumococcal vaccines. This narrative review aims to discuss the evidence to date on the impact of immune-modifying therapies on the immune response to COVID-19 vaccines, with a focus on the first two vaccine doses.

***Recent Findings*:**

Individuals receiving immune-modifying therapy are more likely to have attenuated humoral responses to a single dose of COVID-19 vaccine compared to healthy controls; however, this may be improved by a complete course of vaccination. B cell targeted biologics such as rituximab markedly impair the humoral response to both the first and second COVID-19 vaccination. There remains a paucity of data on cellular immune responses, with the few available studies indicating lower responses to two vaccine doses in individuals receiving immune-modifying therapies compared to healthy controls, which may impact the durability of immune responses.

***Summary*:**

Inadequate humoral immune responses to a single dose of vaccine in the context of immune-modifying therapy are improved by a complete course of vaccination. Individuals receiving immune-modifying treatments should be encouraged to take up a complete vaccine course to mitigate their risk against COVID-19. Research in large patient populations on the longevity/kinetics of the complex humoral and cellular response to subsequent vaccine doses, including against newer variants of concern, is warranted, in addition to data on immune correlates of vaccine clinical effectiveness.

## Introduction

Immune-modifying therapies have improved clinical and quality of life outcomes of individuals with immune-mediated skin diseases such as psoriasis, atopic dermatitis and immunobullous disease. However, these medications can also impair protective immune responses [1, 2]. COVID-19 vaccination programmes have seen great success, with reassuring safety data and two doses of COVID-19 vaccines reported to be 89–97% effective against the development of symptomatic infection in the general population (data pertain to older SARS-CoV-2 variants such as Alpha, Beta, Delta, Gamma) [3]. Given that infection-acquired immunity is limited in duration and susceptible to variants, vaccines are important for mitigating against severe COVID-19 outcomes [4, 5]. Due to concerns over the effectiveness of vaccines in individuals who are receiving immune-modifying therapies, the impact of these medications on the immune response to COVID-19 vaccinations has been an area of intensive investigation [6].

Immune-modifying therapy can be divided into two broad groups: standard systemic and targeted. Standard systemic drugs include methotrexate, ciclosporin, azathioprine, mycophenolate mofetil, acitretin and fumaric acid esters. Targeted therapies used in dermatology include biologics such as TNF-inhibitors (TNFi: adalimumab, certolizumab pegol, etanercept, infliximab), IL-17 inhibitors (IL-17i: bimekizumab, brodalumab, ixekizumab, secukinumab), IL-23 inhibitors (IL-12/IL-23p40 inhibitor ustekinumab, IL-23p19 inhibitors: guselkumab, risankizumab, tildrakizumab), IL-4/IL-13 inhibitor dupilumab and CD20 inhibitor rituximab. Other targeted therapies include small molecule agents such as JAK inhibitors (JAKi: abrocitinib, baricitinib, upadacitinib) and phosphodiesterase inhibitors such as apremilast.

The UK was the first country to approve the use of COVID-19 vaccines in December 2020. Currently there are at least 37 different COVID-19 vaccines approved in different countries worldwide, with more in the development pipeline [7]. In the UK, this includes the BNT162b2 Pfizer/BioNTech, mRNA-1273 Moderna and the ChAdOx1-S Oxford/AstraZeneca COVID-19 vaccines (Table 1) [8]. In the USA, the BNT162b2 Pfizer/BioNTech, mRNA-1273 Moderna, the Janssen Ad26.COV2-S (recombinant) and Novavax COVID-19 vaccines are licensed for use [9, 10]. Both the BNT162b2 Pfizer/BioNTech and mRNA-1273 Moderna vaccine are modified messenger RNA (mRNA) based-vaccines, which utilise lipid nanoparticles to facilitate entry into host cells. Upon entering host cells, the nanoparticle inserts its mRNA into the cytoplasm, where it is translated into the SARS-CoV-2 structural surface glycoprotein (spike [S] protein), which then stimulates humoral and cellular immune responses [11]. The ChAdOx1-S Oxford/AstraZeneca vaccine comprises a replication-deficient chimpanzee adenoviral vector (ChAdOx1), which contains a gene encoding the S protein [12]. The Janssen Ad26.COV2-S COVID-19 vaccine uses a recombinant, replication-incompetent human adenovirus type 26 vector to deliver a gene encoding the S protein. The Novavax COVID-19 vaccine is a protein subunit-based vaccine that comprises recombinant S protein nanoparticle combined with Matrix-M adjuvant [10].

**Table 1:** COVID-19 vaccines approved in the UK and/or the USA

Vaccine	Vaccine platform	Mechanism of action	Posology for general population
BNT162b2 Pfizer/BioNTech	mRNA based	Lipid nanoparticles enter host cells and insert mRNA into the cytoplasm. Translation of S protein stimulates both humoral and cellular immune responses	Primary vaccination course: 2 doses, at least 8 weeks apart Booster/reinforcing/third dose is recommended at least 3 months after the primary course
mRNA-1273 (Moderna)	mRNA based	Lipid nanoparticles enter host cells and insert mRNA into the cytoplasm. Translation of S protein stimulates both humoral and cellular immune responses	Primary vaccination course: 2 doses, at least 8 weeks apart Booster/reinforcing/third dose is recommended at least 3 months after the primary course
ChAdOx1-S Oxford/AstraZeneca	Deficient chimpanzee adenovirus	Replication-deficient chimpanzee adenovirus contains DNA encoding S protein. The DNA is released into the cytoplasm, migrates into the cell nucleus and host enzymes transcribe mRNA, which migrates back to the cytoplasm. Here, it interacts with host cell ribosomes to enable S protein translation	Primary vaccination course: 2 doses, at least 8 weeks apart Booster/reinforcing/third dose is recommended at least 3 months after the primary course
Janssen Ad26.COV2-S	Deficient adenovirus-26	Replication-incompetent recombinant human adenovirus type 26 vector contains DNA encoding S protein. Host enzymes in the cell nucleus transcribe mRNA, which then migrates to the cytoplasm, where S protein translation takes place	Primary vaccination course: 1 dose Booster/reinforcing/second dose of Janssen COVID-19 vaccine not recommended by US Advisory Committee on Immunization Practices due to reports of thrombosis with thrombocytopenia syndrome (TTS) [9]. Preferential use of mRNA COVID-19 vaccines recommended over the Janssen COVID-19 vaccine for primary and booster vaccination in all individuals aged ≥ 18 years in the USA [9]
Novavax COVID-19 Vaccine, Adjuvanted	Subunit protein	Recombinant S protein nanoparticle combined with Matrix-M adjuvant	Primary vaccination course: 2 doses, at least 3 weeks apart Booster/reinforcing/third dose not yet approved

## The Impact of Immune-Modifying Therapy on the Immune Response to COVID-19 Vaccines

There has been longstanding uncertainty and concern over the impact of immune-modifying therapies on the effectiveness of different vaccines; for example, it is well recognised that methotrexate impairs humoral responses to both influenza and pneumococcal vaccines [13]. Since individuals receiving immune-modifying therapies were considered more vulnerable to adverse COVID-19 outcomes than the general population, they have been prioritised during the global COVID-19 vaccine roll-out. Public health policies across the world also advocated an additional COVID-19 vaccine dose as part of the primary course. Therefore, for individuals receiving immune-modifying therapies, a third vaccine dose (BNT162b2 Pfizer/BioNTech, mRNA-1273 Moderna, or ChAdOx1-S Oxford/AstraZeneca vaccines) is considered part of the primary vaccination course and this should be administered at least 8 weeks after the second dose [8]. A booster dose is then recommended at least 3 months following the third primary dose.

While data on clinical effectiveness of vaccines (i.e. in prevention of COVID-19 transmission or severe disease) requires longer-term population-level follow-up, the initial focus of the research community turned to the impact of immune-modifying therapies on the immune response to the COVID-19 vaccine, i.e. immunogenicity. The immune response to a vaccine involves a complex interplay between the humoral and cellular immune response; hence when assessing a vaccine’s immunogenicity, both humoral and cellular immune responses should be considered [14]. Within the humoral response, seroconversion and neutralising activity can be assessed. Seroconversion is the development of antibodies in the blood serum as a result of vaccination. Following COVID-19 vaccination, seroconversion is assessed by measuring IgG antibodies against the S1 domain of the SARS-CoV-2 S protein. Neutralising antibodies can also be measured which target S1 receptor binding domain (RBD), N-terminal domain (NTD) and S2 region of the S protein. These antibodies block viral entry into host cells by preventing the interaction between the host cell angiotensin-converting enzyme 2 (ACE2) and S protein [15, 16]. Finally, assessment of the cellular response to the vaccine involves the analysis of the frequency and function of T cells which recognise viral proteins indirectly and in association with Major Histocompatibility Complex (MHC) I and II molecules [17].

## Methods: Literature Search Strategy

We performed a search of PubMed and Google Scholar databases on March 10, 2022, for studies that have investigated the effect of immune-modifying therapy for skin diseases on COVID-19 vaccine responses. Search terms included “immune therapy”, “immune-modifying therapy”, “immunosuppressant”, “vaccines”, “COVID-19 vaccines”, “COVID-19”, “dermatology” and “skin”, of which “COVID-19”, “COVID-19 vaccines”, “immunosuppressants”, “vaccines”, “skin” and “dermatology” are MESH terms. Outcomes that were analysed included the humoral (seroconversion and/or neutralisation) and cellular responses to the first and second doses of COVID-19 vaccinations. Studies that included individuals with non-skin immune-mediated inflammatory diseases (IMIDs) such as rheumatoid arthritis were included due to shared immune-modifying treatments. There was no minimum size of study and only papers written in English were included.

## Results: Summary of Findings from Literature Search

**Fig. 1:**
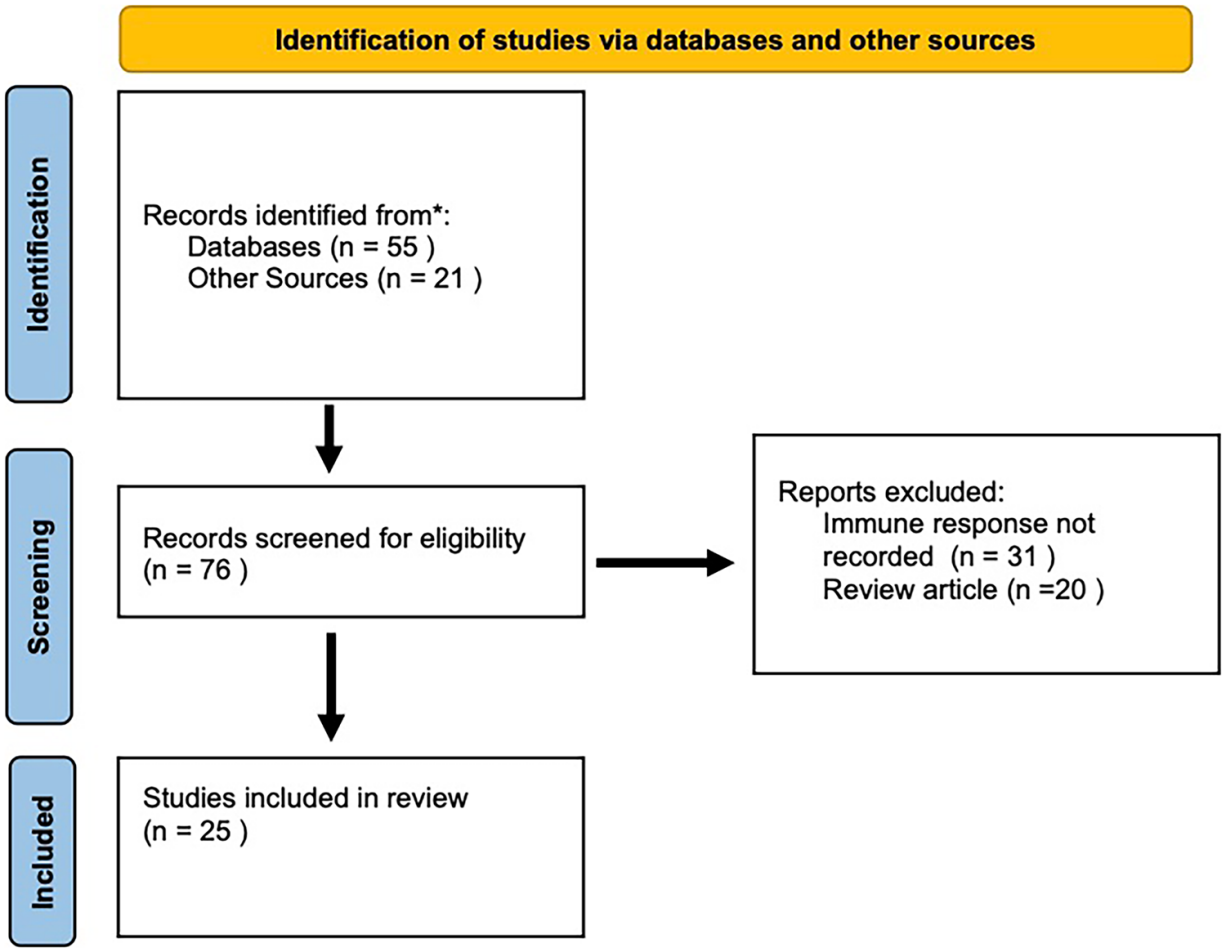
PRISMA flowchart of literature search strategy

Twenty-five studies were identified (Fig. 1, Table 2) [18–32, 33•, 34•, 35–42].

**Table 2:** Details of studies included in this review

**Study**	**Study population (number of individuals, *n*)**	**Demographics**	**Immune-modifying therapy**	**COVID-19 vaccine**	**Immune response after first dose of COVID-19 vaccine**	**Immune response after second dose of COVID-19 vaccine**
**Al-Janabi et al.** [18]	IMIDs (n = 120) including psoriasis, PsA, RA, SLE and Crohn’s disease No controls	**Median age:** 53 years **Gender:** 49 (41%) female **Ethnicity** 111 (92.5%) White, 9 (7.5%) Asian	**Standard systemic** Apremilast, ciclosporin, dimethyl fumarate, methotrexate and prednisone **Targeted** Abatacept, adalimumab, brodalumab, certolizumab, etanercept, guselkumab, ixekizumab, risankizumab, secukinumab, tildrakizumab and ustekinumab	BNT162b2 (60/120, 50%) ChAdOx1 nCoV-19 (60/120, 50%)	**Humoral** **Seroconversion** 23/31 (74%) patients on standard immune-modifying therapy had detectable antibodies vs 73/81 (90%) on targeted immune-modifying therapy at 34 days post-vaccine Use of standard immune-modifying therapy reduced the odds of a detectable antibody response vs targeted immune-modifying therapy (adjusted OR 0.31)	**N/A**
**Ammitzbøll et al.** [19]	SLE (*n* = 61) and RA (*n* = 73) No controls	**Median age:** **SLE – **60 years **RA – **70 years **Gender:** **SLE – **41 (77.1%) female **RA – **49 (67.1%) female **Ethnicity:** Not included	**Standard systemic** Azathioprine, hydroxychloroquine, leflunomide, methotrexate, mycophenolate mofetil, prednisone and salazopurine **Targeted** Abatacept, belimumab, JAKi, IL-6i, rituximab, TNFi	BNT162b2	**N/A**	**Humoral** **Seroconversion** 103/134 (77%) patients had detectable antibodies at 8 days post-second vaccine dose. A lower proportion of patients with RA than SLE had detectable antibodies (49/73 [67%] vs 54/61 [89%]) 4/17 (24%) patients receiving rituximab had detectable antibodies vs 0/6 (0%) receiving rituximab/methotrexate co-therapy
**Boekel et al. ** [20]	IMIDs including RA, PsA, ankylosing spondylitis, axial/peripheral spondyloarthritis, juvenile idiopathic arthritis, SLE, vasculitis, PMR, Sjögren’s disease, multiple sclerosis, sarcoidosis, systemic sclerosis, myositis, mixed connective tissue disease (*n* = 632) Healthy controls (*n* = 289)	**Mean age:** **IMIDs – **63 years **Healthy controls – **63 years **Gender:** **IMIDs – **423 (67%) female **Healthy controls – **195 (67%) female **Ethnicity:** Not included	**Standard systemic** Azathioprine, ciclosporin, hydroxychloroquine, leflunomide, methotrexate, prednisone and sulfasalazine **Targeted** Abatacept, ixekizumab, natalizumab, ocrelizumab, rituximab, secukinumab, tocilizumab, ustekinumab, and TNFi	ChAdOx1 nCoV-19 (514/921, 56%) BNT162b2 (460/921, 50%) mRNA-1273 (55/921 6%) Ad.26.COV2.S (3/921, 0.3%)	**Humoral** **Seroconversion** 210/432 (49%) individuals with IMIDs had detectable antibodies vs 154/210 (73%) healthy controls (adjusted OR 0·33; *p* < 0·0001) at 34 vs 36 days post vaccine, respectively 43/133 (32%) individuals with IMIDs on methotrexate, 1/18 (6%) on rituximab/ocrelizumab, and 24/36 (67%) on TNFi had detectable antibodies	**Humoral** **Seroconversion** 97/106 (92%) individuals with IMIDs had detectable antibodies vs 38/40 (95%) healthy controls at 38 and 42 days post second dose, respectively 17/18 (94%) individuals with IMIDs on methotrexate, 3/7 (43%) on rituximab/ocrelizumab, and 14/14 (100%) on TNFi had detectable antibodies
**Bonelli et al. ** [21]	Rheumatological disease (*n* = 5) including immune-mediated necrotizing myopathy, ANCA-associated vasculitis, eosinophilic granulomatosis, SLE and mixed connective tissue disease Healthy controls: vaccinated (*n* = 4) and unvaccinated (*n* = 4)	**Median age:** **Rheumatological disease – **49 years **Gender:** **Rheumatological disease – **3 (60%) females **Ethnicity:** Not included Demographics for controls not included	**Targeted** Rituximab	BNT162b2	**N/A**	**Humoral** **Seroconversion** 2 of 5 (40%) individuals receiving rituximab had detectable antibodies at 23 days post second vaccination **Cellular** IFN-γ response was detected in the rituximab-treated group (5/5, 100%) and vaccinated healthy control group (4/4, 100%), independent of the humoral immune response
**Boyarsky et al. ** [22]	Rheumatological disease (*n* = 123) including inflammatory arthritis (28%), SLE (20%), Sjogren’s syndrome (13%) and overlap connective tissue diseases (29%) No controls	**Mean age:** 50 years **Gender:** 117 (95%) female **Ethnicity:** 111 (90%) White	**Standard systemic** Azathioprine, hydroxychloroquine, leflunomide, methotrexate, mycophenolate mofetil, sulfasalazine and tacrolimus **Targeted** Abatacept, adalimumab, belimumab, certolizumab, etanercept, infliximab, ixekizumab rituximab, tocilizumab, tofacitinib, ustekinumab	BNT162b2 (64/123, 52%) mRNA-1273 (59/123, 48%)	**Humoral** **Seroconversion** 91/123 (74%) of individuals with rheumatological diseases had detectable antibodies 18–26 days post-vaccine 55/85 (65%) individuals receiving standard immune-modifying therapy had detectable antibodies vs 16/17 (94%) individuals receiving TNFi A lower proportion of individuals receiving rituximab (2/6, 33%) had an antibody response vs those receiving methotrexate (10/13, 77%) or TNFi (16/17, 94%)	**N/A**
**Braun-Moscovici et al. ** [23]	Rheumatological disease **(**n = 264) including RA, PsA, SpA, sarcoidosis, SLE, systemic sclerosis, myositis, Sjogren’s syndrome, myositis and vasculitis Individuals recovered from COVID-19 (*n* = 26)	**Mean age:** **Rheumatological disease – **58 years **Recovered from COVID-19 **– 47 years **Gender:** **Rheumatological disease – **200 (76%) female **Recovered from COVID-19 **– 19 (73%) female **Ethnicity:** Not included	**Standard systemic** Azathioprine, ciclosporin, corticosteroids, hydroxychloroquine, leflunomide, methotrexate, mycophenolate mofetil, Purinethol and salazopurine **Targeted** Abatacept, adalimumab, anakinra, baricitinib, belimumab, certolizumab, etanercept, golimumab, infliximab, ixekizumab, JAKi, mepolizumab, risankizumab, rituximab, sarilumab, secukinumab, tocilizumab, tofacitinib, upadacitinib and ustekinumab	BNT162b2	**N/A**	**Humoral** **Seroconversion** 68/78 (87%) methotrexate-treated individuals, 24/48 (50%) rituximab-treated individuals and 63/63 (100%) individuals on TNFi had detectable antibodies 4–6 weeks following the 2nd dose **Neutralisation** 227/264 (86%) individuals with rheumatological disease had detectable neutralising antibodies 4–6 weeks following the 2nd dose. 23/47 (49%) individuals on rituximab had detectable neutralising antibodies vs 5/8 (63%) individuals on abatacept, 17/21 (81%) individuals on mycophenolate mofetil and 4/5 (80%) individuals on IL-17i. Neutralising titres were higher in vaccinated individuals vs individuals with rheumatological disease who had recovered from COVID-19 (*p* < 0.05)
**Bugatti et al. ** [24]	Rheumatological disease (*n* = 140) including RA, PsA, SpA No controls	**Mean age:** 56 years **Gender:** 95 (67.9%) female **Ethnicity:** Not included	**Standard systemic** Ciclosporin, leflunomide, methotrexate, prednisone and sulfasalazine, **Targeted** Cytotoxic T lymphocyte associated protein-4 immunoglobulin (CTLA4Ig), IL-6Ri, IL-17/IL-23i, JAKi, PDE4i and TNFi	BNT162b2	**Humoral** **Seroconversion** 85/140 (60%) individuals had detectable antibodies after 21 days. Use of methotrexate, glucocorticoids and CTLA4Ig was more common, and use of IL-17/23 inhibitors was less common, in individuals who did not mount detectable antibodies Seroconversion decreased from 85.4% among patients not on methotrexate or glucocorticoids to 33.3% among those on both therapies	**N/A**
**Chen et al. ** [25]	IMIDs including RA, SLE, PsA, multiple sclerosis, ankylosing spondylitis, Sjögren’s syndrome, Hashimoto’s disease, psoriasis, combined variable immune deficiency, vasculitis, scleroderma, ANCA-associated vasculitis, IBD, alopecia areata, asthma and inflammatory arthritis (*n* = 75) Healthy controls (*n* = 25)	**Median age:** 49 years **Gender:** 52 (68%) female **Ethnicity:** 69 (90%) White, 5 (6%) Black, 1 (1%) Asian Demographics for controls not included	**Standard Systemic** Sulfasalazine, antimetabolites, systemic steroids **Targeted** anti-CD20, IL-23i and TNFi	BNT162b2	**N/A**	**Humoral** **Seroconversion** Individuals receiving anti-CD20 had substantially lower levels of spike-specific IgG vs controls at 3 months post second dose. Most other drug treatment groups showed no statistically significant differences in spike-specific IgG titres vs controls **Neutralisation** 48/75 (64%) individuals with IMIDs on immune-modifying therapy developed adequate neutralization titres against Delta variant vs 23/25 (92%) healthy controls at 3 months post vaccine. 8/12 (67%) individuals on TNFi developed adequate neutralization titres compared to 2/9 (22%) on IL-23i and 4/12 (33%) on antimetabolites
**Dailey et al. ** [26]	IBD (*n* = 436) including Crohn’s disease, ulcerative colitis and IBD-unspecified. Only 33 were analysed post vaccination Adult and paediatric controls (number or other details not specified)	**Mean age:** 17 years **Gender:** 183 (42%) female **Ethnicity:** Not included	**Standard systemic** Corticosteroids and methotrexate **Targeted** Infliximab, vedolizumab	BNT162b2 (21/33, 63.6%) mRNA-1273 (7/33, 21.2%) Ad26.COV2-S (5/33, 15.2%)	**N/A**	**Humoral** **Seroconversion** Spike-specific IgG responses were higher (~ 15 ×) following vaccination vs natural infection in the IBD subgroup between 3.1 and 3.3 weeks post 2nd dose (*p* < 0.0001) **Neutralising** All IBD subjects had higher (~ 10 ×) neutralising titres (against SARS-CoV-2 wild type [WT] or mutant [mt S-RBD] spike protein receptor binding domain) post 2^nd^ vaccination vs those with history of natural infection, including patients receiving infliximab monotherapy or infliximab/methotrexate co-therapy
**Deepak et al. ** [27]	IMIDs including RA, spondyloarthritis, uveitis, SLE, vasculitis, Sjogren syndrome, multiple sclerosis and IBD (n = 133) Healthy controls (n = 53)	**Mean age:** **IMIDs – **45 years **Healthy controls – **43 years **Gender:** **IMIDs **– 99 (74.4%) female **Healthy controls **– 29 (54.7%) female **Ethnicity:** **IMIDs **– 117 (88.0%) White, 9 (6.8%) Asian, 4 (3.0%) Black or African American, 3 (2.3%) others **Healthy controls **– 42 (79.2%) White, 7 (13.2%) Asian, 1 (1.9%) Black or African American, 3 (5.7%) other	**Standard systemic** Azathioprine, hydroxychloroquine, leflunomide, methotrexate, mycophenolate mofetil, sulfasalazine and tacrolimus **Targeted** Adalimumab, certolizumab, etanercept, golimumab, guselkumab, infliximab, ocrelizumab, rituximab and ustekinumab	BNT162b2 mRNA-1273 Number of individuals receiving specific vaccines not given	**N/A**	**Humoral** **Seroconversion** 118/133 (89%) individuals with IMIDs vs all healthy controls (53/53, 100%) seroconverted within 14–20 days post-vaccination. No difference between individuals with IMIDs on standard immune-modifying therapy vs those not on standard immune-modifying therapy 6/10 (60%) individuals with IMIDs receiving rituximab/ocrelizumab seroconverted **Neutralising** Neutralisation titres against the common variant (D614G) were 2391 (95% CI 1537 to 3719) in individuals with IMIDs on standard immune-modifying therapy vs 2270 (95% CI, 1650 to 3121) in those not on standard therapy at 20 days post-vaccination Individuals with IMIDs on rituximab/ocrelizumab had lower neutralisation titres vs individuals with IMIDs who were not on rituximab/ocrelizumab (neutralisation titres 723 (95% CI 233 - 2246) vs 2445 (95% CI 1890 - 3164), respectively)
**Furer et al. ** [28]	Rheumatological disease including RA, PsA, axial spondyloarthritis, SLE, systemic vasculitis, large vessel vasculitis (n = 686) Healthy controls (*n* = 121)	**Median age:** **Rheumatological disease **– 59 years **Healthy controls **– 50 years **Gender:** **Rheumatological disease – **475 (69.3%) female **Healthy controls – **78 (65%) female **Ethnicity:** Not mentioned	**Standard systemic** Glucocorticoids, hydroxychloroquine, leflunomide, methotrexate and mycophenolate mofetil **Targeted** Abatacept, IL-6i, IL-17i, JAKi, rituximab and TNFi	BNT162b2	**N/A**	**Humoral** **Seroconversion** 590/686 (86%) patients with rheumatological diseases seroconverted vs 100% controls (*p* < 0.0001), 2–6 weeks after the second vaccination Compared to individuals receiving biologic monotherapy, those on methotrexate monotherapy or methotrexate in combination with other treatments had reduced seroconversion rates (> 97% vs 92%, *p* = 0.02 or 84%, *p*** < **0.0001, respectively) Rituximab impaired seroconversion (39%) Spike specific IgG titres were significantly lower in individuals with rheumatological diseases compared with controls
**Geisen et al. ** [29]	IMIDs including psoriasis, IBD, SLE, RA, spondyloarthropathy, PsA (*n* = 26) Healthy controls (*n* = 42)	**Mean age:** **IMIDs **– 51 years **Healthy controls **– 38 years **Gender:** **IMIDs – **17 (64.3%) female **Healthy controls **– 29 (69.2%) female **Ethnicity:** Not included	**Standard systemic** Azathioprine, leflunomide, hydroxychloroquine and sulfasalazine **Targeted** Adalimumab, belimumab, certolizumab, etanercept, golimumab, infliximab, ixekizumab, secukinumab, tocilizumab, ustekinumab and vedolizumab	BNT162b2 (63/68, 92.6%) mRNA-1273 (5/68, 7.4%)	**N/A**	**Humoral** **Seroconversion** Spike specific IgG was detected in all individuals Lower titres were detected in individuals with IMIDs vs healthy controls (mean titre 2053 BAU/mL vs 2685 BAU/mL, p = 0.037) at 7 days post- second dose. No significant difference between standard immune-modifying therapy, TNFi and IL-17i **Neutralising** Individuals with IMIDs on immune-modifying therapy had lower levels of neutralising antibodies vs healthy controls (mean inhibitory level 87.42% vs 96.04%, *p* = 0.044)
**Haberman et al. ** [30] New York Cohort	IMIDs including psoriasis, PsA, RA, vasculitis, dermatomyositis, adult-onset Still’s disease, sarcoidosis, PMR (*n* = 51) Healthy controls (*n* = 26) New York Cohort	**Mean age:** **IMIDs**, **not on methotrexate **– 49 years **IMIDs, on methotrexate – **63 years **Healthy controls **– 49 years **Gender:** **IMIDs, not on methotrexate – **18 (69.2%) female **IMIDs on methotrexate – **18 (66.7%) female **Healthy controls – **16 (61.5%) female **Ethnicity:** **IMIDs, not on methotrexate – **20 (76.9%), 2 (7.7%) Black, 3 (11.5%) Asian, 3 (11.5%) Hispanic **IMIDs, on methotrexate – **17 (63%) White, 3 (11.1%) Black, 3 (11.1%) Asian, 5 (18.5%) Hispanic **Healthy controls – **16 (61.5%) White, 1 (3.8%) Black, 9 (34.6%) Asian, 1 (3.8%) Hispanic	**Standard systemic** Hydroxychloroquine, leflunomide, methotrexate, oral steroids, sulfasalazine **Targeted** Abatacept, apremilast, IL-17i, IL-23i, JAKi, rituximab and TNFi	BNT162b2	**N/A**	**Humoral** **Seroconversion** 18/25 (72.0%) of IMID cohort on methotrexate had detectable antibodies vs 24/26 (92.3%) not on methotrexate (p = 0.045). 25/26 (96.1%) healthy controls had detectable antibodies Timing of sampling not specified **Cellular** Spike-specific B cells, activated CD4 + T cells and circulating T follicular helper cells were similarly induced in all groups Activated CD8+ T-cell responses were attenuated in methotrexate-treated group vs healthy controls or individuals with IMIDs not receiving methotrexate
**Haberman et al. ** [30] Erlangen cohort	IMIDs including psoriasis, PsA, RA, vasculitis, dermatomyositis, adult-onset Still’s disease, sarcoidosis, PMR (n = 31) Healthy controls (*n* = 182) Erlangen cohort	**Mean age:** **IMIDs, not on methotrexate **– 45 years **IMIDs, on methotrexate – **55 years **Healthy controls – **41 years **Gender:** **IMIDs, not on methotrexate **– 7 (58.3%) female **IMIDs, on methotrexate – **15 (75.0%) female **Healthy controls – **104 (57.1%)** female** **Ethnicity:** **IMIDs, not on methotrexate–**11 (100%) White **IMIDs, on methotrexate – **19 (95%) White and 1 (5%) Asian **Healthy controls – **178 (97.8%) White, 2 (1.1%) Asian, 1 (0.6%) Hispanic	**Standard systemic** Methotrexate **Targeted** TNFi	BNT162b2	**N/A**	**Humoral** **Seroconversion** 10/11 (90.9%) patients not on methotrexate, and 10/20 (50.0%) patients receiving methotrexate had detectable antibodies vs 179/182 (98.3%) healthy controls Timing of sampling not specified
**Kappelman et al. ** [31]	IBD (*n* = 317) No controls	**Mean age:** 51 years **Gender:** 238 (75%) female **Ethnicity:** 301 (95%) White, 5 (2%) Asian	**Standard systemic** 6-mercaptopurine, azathioprine, budesonide, methotrexate and sulfasalazine **Targeted** TNFi, ustekinumab and vedolizumab	BNT162b2 (173/317, 55%) mRNA-1273 (144/317, 45%)	**N/A**	**Humoral** **Seroconversion** 300/317 (94.6%) had detectable antibodies at 64 days post-2nd vaccination. 11/13 (85%) individuals taking corticosteroids had detectable antibodies vs 289/304 (95%) non-steroid users 19/20 (95%) individuals on standard immune-modifying therapy had detectable antibodies vs 101/108 (94%) individuals on TNFi monotherapy
**Kennedy et al. ** [32]	IBD including Crohn’s disease, ulcerative colitis or IBD unclassified who have received one dose of vaccination (*n* = 1293) and two doses (*n* = 27) No healthy controls	**Mean age:** **Overall – **44 years **Infliximab **– 41 years **Vedolizumab – **49 years **Gender:** **Overall – **634 (49.2%) female **Infliximab – **434 (50.3%) female **Vedolizumab – **200 (47.1%) female **Ethnicity:** **Overall – **1172 (91.1%) White, 78 (6.1%) Asian, 24 (1.9%) mixed, 8 (0.6%) Black **Infliximab – **791 (91.8%) White, 46 (5.3%) Asian, 16 (1.9%) mixed, 6 (0.7%) Black **Vedolizumab – **381 (89.9%) White, 32 (7.5%) Asian, 8 (1.9%) mixed, 2 (0.5%) Black	**Targeted** Infliximab and vedolizumab	BNT162b2 (589/1293, 45.6%) ChAdOx1 nCoV-19 (704/1293, 54.4%)	**Humoral** **Seroconversion** Spike-specific IgG titres were lower in patients treated with infliximab vs vedolizumab, at 3–10 weeks following BNT162b2 (6.0 U/mL vs 28.8 U/mL, *p* < 0.0001) or ChAdOx1 nCoV-19 (4.7 U/mL vs 13.8 U/mL, *p* < 0.0001)	**Humoral** **Seroconversion** 17/20 (85%) infliximab-treated individuals and 6/7 (86%) vedolizumab-treated individuals seroconverted at 3–10 weeks post-vaccination (*p* = 0.68)
**Mahil et al.** [33•]	Psoriasis (n = 84) Healthy controls (n = 17)	**Median age:** **Methotrexate **– 48 years **TNFi–**36 years **IL-17i – **45 years **IL-23i – **50 years **Healthy controls – **34 years **Gender:** **Methotrexate **– 6 (35%) female **TNFi – **14 (52%) female **IL-17i – **7 (47%) female **IL-23i – **10 (40%) female **Healthy controls – **8 (47%) female **Ethnicity:** **Methotrexate – **13 (76%), 1 (6%) Black, 3 (18%) South Asian **TNFi – **24 (89%), 3 (11%) South Asian **IL-17i – **13 (87%) White, 2 (13%) South Asian **IL-23i – **21 (84%) White, 3(12%) South Asian, 1 (4%) mixed **Healthy controls – **14 (82%) White, 3 (18%) South Asian	**Standard systemic** Methotrexate **Targeted** Adalimumab, certolizumab, etanercept, guselkumab, infliximab, ixekizumab, risankizumab secukinumab and ustekinumab	BNT162b2	**Humoral** **Seroconversion** 60/77 (78%) individuals receiving immune-modifying therapy vs 17/17 (100%) healthy controls seroconverted, at 28 days post-vaccine Individuals receiving methotrexate (7/15, 47%) had a lower seroconversion rate vs individuals receiving biologics including TNFi (19/24, 79%), IL-17i (15/15, 100%) and IL-23i (19/23, 83%) **Neutralising** Individuals receiving TNFi, IL-17i, and IL-23i had similar neutralisation titres against wild-type SARS-CoV-2 vs controls. Patients on methotrexate had lower neutralisation titres (median ID_50_ 129 [IQR 40–236]) vs patients on biologics (269 [141–418], p = 0·011) or healthy controls (317 [213–487], p = 0.0032) **Cellular** T-cell response rates were similar in patients receiving methotrexate, biologics, and controls at 28 days post-vaccine	**N/A**
**Mahil et al.** [34•]	Psoriasis (*n* = 67) Healthy controls (*n* = 15)	**Median age:** **Methotrexate – **50 years **TNFi – **36 years **IL-17i – **43 years **IL-23i – **51 years **Healthy controls – **38 years **Gender:** **Methotrexate – **6 (43%) female **TNFi – **11 (58%) female **IL-17i – **7 (50%) female **IL-23i – **8 (40%) female **Healthy controls – **7 (47%) female **Ethnicity:** **Methotrexate – **11 (79%) White, 1 (7%) Black, 2 (14%) South Asian **TNFi – **18 (95%) White, 1 (5%) South Asian **IL-17i – **12 (86%) White, 2 (14%) South Asian **IL-23i – **17 (85%) White, 2 (10%) South Asian, 1 (5%) mixed **Healthy controls – **13 (87%) White, 2 (13%) South Asian	**Standard systemic** Methotrexate **Targeted** IL-17i, IL-23i and TNFi	BNT162b2	**N/A**	**Humoral** **Seroconversion** All participants seroconverted 14 days post-second vaccine The second vaccine boosted spike-specific IgG titres in all groups, with no significant differences between patients receiving immune-modifying therapy (median EC_50_ 1816 [IQR 641–3645]) vs healthy controls (2749 [867–4770], *p* = 0·14). Median titres were lowest in individuals on methotrexate (median EC_50_ 1751 [IQR 468–4976]) vs patients on biologics (1816 [787–3534], *p* = 0·65) or healthy controls (2749 [867–4770], *p* = 0·20) **Neutralising** All participants had detectable neutralising antibodies against wild-type, Alpha, and Delta variants post-second vaccine. All groups showed increased neutralising antibody titres post-second vs first dose. Individuals on methotrexate had similar neutralisation activity against the Alpha variant (ID_50_ 440 [IQR 101–935]) vs those receiving biologics (453 [233–955], *p* = 0·97) or healthy controls (491 [264–1227], *p* = 0·63). Neutralisation activity against the Delta variant was similar across all groups i.e. individuals on methotrexate (ID_50_ 856 [77–1476]), biologics (469 [232–1309], *p* = 0·76) and controls (863 [225–1297], *p* = 0·49) **Cellular** Those on methotrexate had lower induction of T-cell responses vs healthy controls. A lower proportion of patients receiving methotrexate had detectable T-cell responses (8/13, 62% [95%CI 32–86]) vs those on biologics (37/50, 74% [95%CI 60–85], *p* = 0·38) or controls (14/14, 100% [95%CI 77–100], *p* = 0·022)
**Moor et al. ** [35]	Patients including ANCA-associated vasculitis, RA, Sjogren’s syndrome, SLE, multiple sclerosis, pemphigus vulgaris, systemic sclerosis and pemphigoid (*n* = 96) Healthy controls (*n* = 29)	**Median age:** **Patients – **67 years **Healthy controls – **54 years **Gender:** **Patients – **51 (53%) female **Healthy controls – **19 (66%) female **Ethnicity:** Not included	**Targeted** Ocrelizumab or rituximab	BNT162b2 (58/96, 60%) mRNA-1273 (38/96, 40%)	**N/A**	**Humoral** **Seroconversion** Detectable antibodies in 47/96 (49%) patients on rituximab/ocrelizumab at 1·79 months post-second vaccine dose, vs 29/29 (100%) controls at 1·81 months (*p* < 0·001) **Cellular** SARS-CoV-2-specific IFNγ responses detected in 14/44 (32%) patients on rituximab/ocrelizumab vs 22/25 (88%) healthy controls (*p* < 0·001)
**Rubbert-Roth et al. ** [36]	RA (n = 53) Healthy controls (n = 20)	**Mean age:** **RA – **65 years **Healthy controls – **45 years **Gender:** **RA – **29 (54.7%) female **Healthy controls – **14 (70%) female **Ethnicity:** Not included	**Standard systemic** Leflunomide, methotrexate and prednisone **Targeted** Anti-cytokine drugs (specific drugs not mentioned), abatacept, JAKi	BNT162b2 (64/73, 87.7%) mRNA-1273 (9/73, 12.3%)	**Humoral** **Seroconversion** 5/51 (10%) individuals with RA on immune-modifying therapy had detectable antibodies post-vaccine vs 18/20 (90%) controls (*p* < 0·001) Spike specific IgG titres were lower in patients with RA on immune-modifying therapy (median 0.4 U/ml, IQR 0.4–2.13) vs healthy controls (99.2 U/ml, 24.8–172, *p* < 0.0001) at 3 weeks post-vaccination	**Humoral** **Seroconversion** Spike specific IgG titres were lower in individuals with RA (657 U/ml, IQR 188–2500) vs healthy controls (2500 U/ml, IQR 2500–2500, *p* < 0.0001) at 2 weeks after the second vaccination 45/51 (88%) individuals with RA on immune-modifying therapy had detectable antibody titres post-second vaccine. Of 6 individuals who did not have detectable antibodies, 4 were receiving JAKi monotherapy or combination therapy, 1 methotrexate/prednisolone co-therapy and 1 methotrexate/prednisolone co-therapy. **Neutralising** A neutralising humoral response was detected in 13/16 (81%) individuals on standard systemic therapy, 17/18 (94%) on cytokine targeted biologic therapy, 4/5 (80%) on abatacept, and 8/12 (67%) on JAKi therapy
**Ruddy et al. ** [37]	Rheumatological disease (n = 404) including RA, ankylosing spondylitis, PsA, reactive arthritis, SLE, Sjogren’s syndrome, myositis, systemic sclerosis and vasculitis No controls	**Median age:** 44 years **Gender:** 385 (96%) female **Ethnicity:** 367 (91%) White	**Standard systemic** Azathioprine, ciclosporin, dimethyl fumarate, hydroxychloroquine, leflunomide, methotrexate, mycophenolate mofetil, sirolimus, sulfasalazine and tacrolimus **Targeted** Abatacept, anakinra, belimumab, guselkumab, IL-6i, IL-17i, JAKi, rituximab, TNFi and ustekinumab	BNT162b2 (198/404, 49%) mRNA-1273 (204/404, 51%)	**N/A**	**Humoral** **Seroconversion** 378/404 (94%) of individuals with rheumatological disease seroconverted at a median of 29 days after 2nd dose A greater proportion of those receiving TNFi had detectable antibodies (100%), vs those receiving mycophenolate (73%, *p* < 0.001), rituximab (26%, *p* < 0.001) or glucocorticoids (82%, *p* < 0.001) or those with a diagnosis of myositis (79%, *p* = 0.01) Lower median antibody titres in individuals receiving mycophenolate mofetil (8 U/mL) and rituximab (< 0.4 U/mL) vs individuals receiving glucocorticoid monotherapy (> 250 U/mL)
**Seree-aphinan et al. ** [38]	Cases: Individuals with pemphigus, psoriasis and chronic spontaneous urticaria who are receiving immune-modifying therapy (*n* = 14) Controls: individuals (with acne, melasma, androgenetic alopecia, seborrheic keratosis) who are not receiving immune-modifying therapy (*n* = 18)	**Mean age:** **Controls – **45 years **Cases – **44 years **Gender:** **Controls – **11 (61%) female **Cases – **10 (71%) female **Ethnicity:** Not included	**Standard systemic** Azathioprine, ciclosporin, methotrexate, mycophenolate mofetil and prednisone **Targeted** Ixekizumab, omalizumab and secukinumab	CoronaVac (Sinovac)	**N/A**	**Humoral** **Seroconversion** No individuals (0/6, 0%) taking azathioprine, ciclosporin, mycophenolate mofetil, or moderate-to-high dose corticosteroids seroconverted 4 weeks post-second vaccine. 7/8 (87.5%) individuals on low dose methotrexate, low dose systemic corticosteroids, or biologics seroconverted **Neutralising** Patients on azathioprine, ciclosporin, mycophenolate mofetil, or moderate-to-high dose corticosteroids had lower neutralizing activity vs those receiving low dose methotrexate, low dose systemic corticosteroids or biologics
**Seyahi et al. ** [39]	IMIDs (*n* = 104) including RA, SLE, Sjogren’s syndrome, polymyositis, ankylosing spondylitis, PsA or psoriasis, Behcet’s syndrome, vasculitis, Familial Mediterranean Fever, IBD. This cohort comprises hospital workers (*n* = 82) and elderly individuals aged 65 and above (*n* = 22) Healthy controls (*n* = 347), comprising hospital workers (*n* = 300) and elderly individuals (*n* = 47)	**Mean age:** **Hospital workers with IMIDs – **42 years **Elderly group with IMIDs – **71 years **Control hospital workers – **71 years, **Control elderly group – **71 years **Gender:** **Hospital workers with IMIDs – **53 (64.6%) female **Elderly group with IMIDs – **16 (72.7%) female **Control hospital workers – **193 (64.3%) female **Control elderly group – **24 (51.1%) female **Ethnicity:** Not included	**Standard systemic** Azathioprine, leflunomide, methotrexate mycophenolate mofetil, prednisone, sulfasalazine and tacrolimus **Targeted** Anakinra, interferon-alpha, rituximab, secukinumab, TNFi and tocilizumab	CoronaVac (Sinovac)	**N/A**	**Humoral** **Seroconversion** A lower proportion of hospital workers with IMIDs had detectable antibodies vs hospital worker controls (92.7% vs 99.7%, *p* < 0.001) at least 21 days following the second vaccination. A lower proportion of elderly individuals with IMIDs had detectable antibodies vs elderly controls (77.3% vs 97.9%, *p* = 0.011) at least 21 days post-second vaccine A lower proportion of individuals receiving rituximab (1/7, 14.3%) had detectable antibodies vs those receiving other targeted therapy (22/25, 88%), standard therapy (25/27, 92.6%) or no treatment (29/29, 100%)
**Simon et al. ** [40]	IMIDs including SpA, RA, IBD, psoriasis, SLE, systemic sclerosis, IgG4-related diseases, GCA, periodic fever syndromes, granulomatosis with polyangiitis and polymyalgia rheumatica (*n* = 84) Healthy controls (*n* = 182)	**Mean age:** **IMIDs – **53 years **Healthy cohort –** 41 years **Gender:** **IMIDs – **55 (65.5%) female **Healthy controls–**104 (57.1%) female **Ethnicity:** Not included	**Standard systemic** Hydroxychloroquine, methotrexate, sulfasalazine **Targeted** IL-17/23i, JAKi and TNFi	BNT162b2	**Humoral** **Seroconversion** 79/84 (94%) individuals with IMIDs on immune-modifying therapy vs 182/182 controls (100%) had detectable antibodies at 11 days post-vaccine (*p* = 0.003)	**Humoral** **Seroconversion** 79/84 (94%) individuals with IMIDs on immune-modifying therapy vs all (100%) controls had detectable antibodies up to 39 days post-second vaccine (*p* = 0.003). No difference between individuals with IMIDs on standard or targeted therapy or no treatment **Neutralising** 76/84 (90.5%) individuals with IMIDs on immune-modifying therapy vs 181/182 (99.5%) controls had neutralising antibodies (*p* = 0.0008)
**Wieske et al. ** [41]	IMIDs on immune-modifying therapy, including vasculitis, Sjogren’s syndrome, PMR, GCA, vitiligo, pemphigus, psoriasis, auto-immune hepatitis, auto-immune sclerosing cholangitis, chronic inflammatory demyelinating polyneuropathy, multifocal motor neuropathy, inflammatory myositis, neuromyelitis optica spectrum disorder (*n* = 1692) IMIDs not on immune-modifying therapy (*n* = 419) Healthy controls (*n* = 174)	**Mean age:** 50 years overall **Gender:** 1470 (62.8%) female overall **Ethnicity:** Not included	**Standard systemic** Ciclosporin, hydroxychloroquine, methotrexate, mycophenolate mofetil and sulfasalazine **Targeted** Abatacept, anti-CD20, belimumab, dupilumab, JAKi, IL-17i, IL-23i, interferon-beta, natalizumab, omalizumab, tocilizumab, TNFi, ustekinumab and vedolizumab **Others:** Intravenous or subcutaneous immunoglobulin, purine antagonists, and sphingosine-1-phosphate receptor modulators	ChAdOx1 nCoV-19 (254/2339, 11%) BNT162b2 (1324/2339, 57%) mRNA-1273 (712/2339, 30%) Ad.26.COV2.S (49/2339, 2%)	**N/A**	**Humoral** **Seroconversion** 39/128 (30·5%) on anti-CD20 therapy seroconverted vs 479/493 (97·2%) controls Compared with controls, relative risk of seroconversion post-second vaccine on anti-CD20 therapy was 0.32 (95% CI 0·19–0·49), while the relative risks for other immune-modifying drugs were not significantly reduced For those who seroconverted, anti-CD20 was associated with substantial reductions in antibody titres. TNFi, dupilumab, intravenous and subcutaneous immunoglobulin, and methotrexate were associated with moderate reductions in antibody titres **Neutralisation** Neutralisation capacity of individuals on immune-modifying monotherapy was similar to controls
**Wong et al. ** [42]	IBD (*n* = 48) Vaccinated healthcare workers (HCWs, *n* = 14) Healthy controls (*n* = 29)	**Mean age:** **IBD – **49 years **HCWs – **35 years **Healthy controls – **32 years **Gender:** **IBD – **25 (52%) female **HCWs -**7 (50%) female **Healthy controls –** 11 (37.9%) female **Ethnicity:** **IBD – **42 (87.5%) White **HCWs – **10 (71.4%) White **Healthy controls – **18 (62.1%) White	**Targeted** Guselkumab, TNFi, vedolizumab and vedolizumab with thiopurine	BNT162b2 (54/91, 59.3%) mRNA-1273 (37/91, 40.7%)	**N/A**	**Humoral** **Seroconversion** 26/26 (100%) individuals with IBD on targeted therapy had detectable antibodies at 8–18 days post-second vaccine. Vedolizumab was associated with lower spike specific IgG titres vs controls (*p* = 0.0043)

*IMIDs* immune-mediated inflammatory diseases, *PsA* psoriatic arthritis, *RA* rheumatoid arthritis, *SLE* systemic lupus erythematosus, *TNFi* tumour necrosis factor inhibitor, *JAKi* Janus kinase inhibitor, *IL-6i* interleukin-6 inhibitor, *ANCA* antineutrophil cytoplasmic antibodies, *PMR* polymyalgia rheumatica, *IFN-γ* interferon-γ, *IL-17i* interleukin-17 inhibitor, *SpA* spondyloarthritis, *IL-23i* interleukin-23 inhibitor, *PDE4i* phosphodiesterase 4 inhibitor, *IQR* interquartile range, *95% CI* 95% confidence interval, *IBD* inflammatory bowel disease, *GCA* giant cell arteritis, *HCWs* healthcare workers

## Immune Response to the 1st Dose of COVID-19 Vaccine

Eight of twenty-five included studies investigated the effects of immune-modifying therapy on the immune response to a first dose of COVID-19 vaccine [18, 20, 22, 24, 32, 33•, 36, 40]. All eight studies evaluated the impact of immune-modifying therapy on seroconversion and one study additionally analysed neutralisation and cellular responses [33•]. Assessment of the serological response to vaccination (i.e. IgG antibody specific to the SARS-CoV-2 S protein) is highly represented due to the accessibility and convenience of the antibody assay.

### Seroconversion

#### The Standard Systemic Immune-Modifying Therapy Methotrexate May Impair Seroconversion Following the First Vaccine Dose More Than Targeted Therapies Such As TNF Inhibitors

All 8 studies analysing seroconversion following the first vaccine dose showed that individuals who were receiving immune-modifying therapy were less likely to mount a detectable antibody response compared to healthy controls [18, 22, 24, 32, 33, 36 40]. Use of standard systemic immune-modifying therapies had a greater impact on seroconversion than targeted therapies. For example, Boyarsky et al. found that 16 of 17 (94%) individuals receiving TNF-inhibitor biologic therapy had detectable antibodies, compared to 55 of 85 (65%) individuals receiving standard systemic therapy [22]. Specifically, methotrexate was associated with lower seroconversion rates post-vaccination compared with targeted biologics such as TNF-inhibitors [18, 20, 22, 24, 33•]. For example, Al-Janabi et al. (*n* = 120) found that use of methotrexate was associated with a reduced odds of a detectable antibody response compared with targeted therapies (adjusted odds ratio 0.31) [18]. Bugatti et al. (*n* = 140) showed that seroconversion rates decreased from 85% among individuals who were not receiving methotrexate or glucocorticoids to 33% among those receiving both therapies [24]. Our own study (individuals with psoriasis, *n* = 84; healthy controls, *n* = 17) similarly found that individuals receiving methotrexate had lower seroconversion rates (7/15, 47%) compared to those receiving targeted biologic therapy (53/62, 85%, *p* = 0.0001) or healthy controls (17/17, 100%, *p* = 0.0001) [33•].

#### Rituximab Attenuates Seroconversion Following the First Vaccine Dose Compared to Other Immune-Modifying Therapies

Rituximab has a marked effect on seroconversion after the first vaccine dose: Boyarsky et al. found that a lower proportion of individuals receiving rituximab developed an antibody response (2/6, 33%), compared to individuals receiving TNF-inhibitors (16/17, 94%) or standard systemic therapy including methotrexate (10/13, 77%), leflunomide (2/4, 50%), sulfasalazine (4/5, 80%) and hydroxychloroquine (27/37, 73%) [22].

#### Individuals Receiving Immune-Modifying Therapy Have Lower Median Spike-Specific IgG Titres Following the First Vaccine Dose Compared to Healthy Controls

Individuals receiving either standard or targeted systemic immune-modifying therapy who successfully seroconverted after the first vaccine dose had evidence of lower median antibody titres against S protein compared to healthy controls [34•, 36]. For example, Rubbert-Roth et al. found that antibody titres against S protein at 3 weeks after the first vaccine dose were significantly lower in individuals receiving standard or targeted immune-modifying therapy (median titre 0·4 U/mL [IQR 0.4–2.13], *n* = 53) compared to healthy controls (median titre 99·2 U/mL [IQR 24.8–172], *n* = 20, *p* < 0.0001) [36].

### Neutralisation Response

#### Methotrexate Is Associated with Lower Median Neutralisation Titres Following the First Vaccine Dose Compared to Targeted Immune-Modifying Therapy

In our study of neutralisation response to the first dose of COVID-19 vaccine BNT162b2, individuals receiving targeted biologic therapy (TNFi, IL-17i or IL-23i) had similar neutralisation titres against wild-type or Alpha SARS-CoV-2 compared to healthy controls [33•]. Methotrexate use, however, was associated with lower median neutralisation titres (ID_50_ 129 [IQR 40–236]) compared to either healthy controls (ID_50_ 317 [IQR 213–487], *p* = 0.0032) or patients receiving biologic therapy (ID_50_ 269 [IQR 141–418], *p* = 0·011) [33•].

### Cellular Response

#### Methotrexate or Targeted Immune-Modifying Therapy Does Not Significantly Impair Cellular Responses Following the First Vaccine Dose

Our study also analysed cellular responses, which involved quantifying T cells secreting interferon-γ, IL-2 or IL-21 in response to stimulation with two peptide pools spanning the entire length of the S protein [33•]. Methotrexate or targeted biologics did not impair the cellular response to the first dose of COVID-19 vaccine BNT162b2 as all study groups including the healthy control group showed comparable spike-specific T-cell responses.

## Immune Response to the 2nd Dose of COVID-19 Vaccine

Twenty-one studies analysed the effects of immune-modifying therapy on the immune response to two doses of COVID-19 vaccine [19–21, 23, 25–32, 34•, 35–42]. All studies analysed seroconversion following the second vaccine dose. Ten and four studies additionally analysed neutralisation and cellular responses, respectively.

### Seroconversion

#### The Majority of Individuals Receiving Standard Systemic or Targeted Immune-Modifying Therapy (Except Rituximab) Have Evidence of Successful Seroconversion Following the Second Vaccine Dose

The second COVID-19 vaccine dose led to successful seroconversion in the majority of individuals receiving either standard or targeted immune-modifying therapy (excluding rituximab) [19–21, 23, 25, 27–29, 31, 34•, 35–42]. One study showed that only 10 of 20 (50%) patients with IMIDs who were receiving methotrexate seroconverted compared with 179/182 (98%) healthy controls; however, this involved a limited patient sample, and the mean age of healthy controls (41 years) was lower than those receiving methotrexate (55 years) [30]. Although data are limited, prior studies indicate that JAK inhibitors (JAKi) do not impair seroconversion: Furer et al. and Braun-Moscovici et al. found that 19/21 (90%) and 9/9 (100%) individuals receiving JAKi successfully seroconverted, respectively, after the second vaccine dose [23, 28].

#### Rituximab Impairs Seroconversion After the Second Vaccine Dose Compared to Other Immune-Modifying Therapies

All studies assessing rituximab showed that a lower proportion of individuals receiving rituximab seroconverted after the second vaccine dose compared to those who were receiving standard systemic or other targeted immune-modifying therapy, or healthy controls (at 2–4 weeks post second vaccine dose) [19–21, 23, 25, 27–32, 33•, 34•, 35–39, 41]. Seroconversion rates in individuals receiving rituximab were as low as 14.3% after the second COVID-19 vaccine [39]. Vaccinations were administered between 1.5 and 12 months after rituximab treatment. The degree of B cell recovery at the time of vaccination correlated with the extent of humoral vaccine response; i.e. a more favourable humoral response was achieved when there was a greater time interval between rituximab treatment and vaccination [27, 28]. For example, Furer et al. (*n* = 86 on rituximab) found that 50% of individuals seroconverted when their vaccination was 1-year after rituximab treatment, compared with less than 20% of those who were vaccinated within 6 months after rituximab treatment [28].

#### Individuals Receiving Immune-Modifying Therapy Have Lower Spike-Specific IgG Titres Following the Second Vaccine Dose, Compared to Healthy Controls

Individuals receiving immune-modifying therapy had lower titres of spike-specific IgG compared to healthy controls [28, 29]. For example, Furer et al. identified mean antibody titres of 132.9 BAU/ml in individuals with rheumatological diseases who were receiving immune-modifying therapy (including methotrexate, mycophenolate mofetil, leflunomide, hydroxychloroquine, TNFi, IL-6i, IL-17i, rituximab, abatacept and JAKi, *n* = 686) compared to 218.6 BAU/mL in the healthy control group (*n* = 121, *p* < 0.0001) [28]. Furer et al. also found that use of methotrexate as monotherapy or in combination with other treatments was associated with lower titres of spike-specific IgG compared with controls. Similarly, our study identified lower median spike-specific IgG titres following the second vaccine dose in those receiving methotrexate (EC_50_ 1751 [IQR 468–4976]) compared with those receiving targeted biologics (EC_50_ 1816 [IQR 787–3534], *p* = 0.65) or healthy controls (EC_50_ 2749 [IQR 867–4770], *p* = 0.2), albeit not statistically significant [34•]. Wieske et al. (*n* = 26 on JAKi; *n* = 57 on dupilumab) reported that use of JAKi or dupilumab was associated with a moderate decrease in antibody titres compared to healthy controls (*n* = 174) [41].

Despite emerging research on longer-term antibody responses to COVID-19 vaccines in the general population, there remains a paucity of knowledge on the durability of antibody responses in individuals receiving immune-modifying treatment [43]. Data from the general population highlight that the humoral immune response to the second COVID-19 vaccination wanes over time. For example, one study in Estonia involving 122 infection-naïve individuals without significant comorbidities (age range 21–69 years) who were not receiving immune-modifying therapies showed a progressive decline of spike-specific IgG levels at 12 weeks (5226 AU/mL) and 6 months (1383 AU/mL) following the second COVID-19 vaccine [43]. At 6 months after the second vaccine, spike-specific IgG levels were similar to levels in people who had received only one vaccine dose or in those with previous COVID-19 infection (and no vaccination). A large-scale UK study of individuals infected with the Omicron variant (*n* = 86,774), Delta variant (*n* = 204,154) and uninfected controls (*n* = 1,572,621) found that 2 vaccine doses (ChAdOx1 nCoV-19 or BNT162b2) were more effective at protecting against symptomatic disease arising from the Delta variant compared to the Omicron variant [44]. Vaccine effectiveness waned rapidly, with limited effects detected after 20 weeks of the second dose of either vaccine. These general population data underscore the importance of subsequent vaccine doses, particularly in the context of newer variants of concern.

### Neutralisation Response

#### The Majority of Individuals Receiving Immune-Modifying Therapy Develop a Neutralising Response After the Second Vaccine Dose

In ten studies where neutralisation was investigated [23, 25–27, 29, 34•, 36, 38, 40, 41], the majority of individuals receiving immune-modifying therapy developed a neutralising response after the second vaccine dose (range 63–100%, with the exception of those receiving rituximab); however, neutralisation titres were lower compared to healthy controls in 3 studies [27, 29, 38]. Our own study (individuals with psoriasis, *n* = 67; healthy controls, *n* = 15) showed that all study participants had detectable neutralising antibodies against wild-type, Alpha and Delta SARS-CoV-2 variants following the second vaccine dose, and there were higher neutralising antibody titres after the second dose compared to the first [34•]. Neutralisation activity against the Alpha variant was similar in individuals receiving methotrexate (median ID_50_ 440 [IQR 101–935]) compared to those receiving targeted therapy (median ID_50_ 453 [IQR 233–955], *p* = 0·97) or healthy controls (median ID_50_ 491 [IQR 264–1227], *p* = 0·63). With respect to the Delta variant, neutralisation activity was also detected at similar levels across all groups, i.e. in individuals receiving methotrexate (median ID_50_ 856 [IQR 77–1476] versus those receiving targeted therapy (median ID_50_ 469 [IQR 232–1309], *p* = 0·76) or healthy controls (median ID_50_ 863 [IQR 225–1297], *p* = 0·49) [34•]. In contrast, in a smaller study (*n* = 14), Seree-aphinan et al. found that individuals receiving azathioprine, ciclosporin, mycophenolate mofetil or moderate-to-high dose corticosteroids monotherapy had a lower neutralising response compared to those who received low dose methotrexate or low dose systemic corticosteroids or biologics [38].

#### Rituximab Impairs Neutralisation Responses to the Second Vaccine Dose Compared to Other Immune-modifying Therapies

Patients receiving rituximab were less likely to develop detectable neutralising antibodies after the second vaccine dose compared to controls or those receiving other immune-modifying therapies. For example, Deepak et al. (individuals with IMIDs, *n* = 133; healthy controls, *n* = 53) found that neutralisation titres in individuals with IMIDs (including RA, spondyloarthritis, uveitis, SLE, vasculitis, Sjogren syndrome, multiple sclerosis and IBD) who were receiving anti-CD20 therapy were lower compared with those who were not receiving anti-CD20 therapy (half-maximal dilution titres 723 vs 2445, respectively) [27].

### Cellular Responses

#### Methotrexate May Impair the Cellular Immune Response to the Second Vaccine Dose

In comparison to data available on humoral responses, there is a relative paucity of data on cellular immune responses to the COVID-19 vaccine [45]. This represents an important knowledge gap because studies indicate that a robust cellular response is critical in the protection against severe COVID-19 by enabling accelerated viral clearance [17]. SARS-CoV-2-specific T cell responses may help to prevent infection and may also support the development of robust immunological memory since CD4 spike-specific T cell responses promote antibody generation, with follicular helper T cells correlating with long-term/durable humoral immunity [45]. Furthermore, unlike humoral responses, which seem to be evaded by many variants of concern, cellular responses seem to show stronger cross-protection highlighting their importance in the face of ongoing viral evolution [46].

The four studies identified in this review that investigated cellular responses in individuals receiving immune-modifying therapy yielded broadly similar findings [21, 30, 34•, 35]. For example, our own study found that a lower proportion of individuals receiving methotrexate (8/13 [62%], 95% CI 32–86) had detectable T-cell responses following the second vaccine dose compared to those receiving targeted biologics (37/50 [74%], 95% CI 60–85; *p* = 0·38) or controls (14/14 [100%], 95% CI 77–100%, *p* = 0·022) [34•]. Similarly, Haberman et al. (individuals with IMIDs, *n* = 51; healthy controls, *n* = 26) reported attenuated activated CD8+ T-cell responses in methotrexate-treated individuals following the second COVID-19 vaccine dose compared to healthy controls or individuals with IMIDs not on methotrexate [30]. Of note, Moor et al. found that individuals receiving B cell targeted biologics (rituximab or ocrelizumab) also had reduced T cell responses compared to healthy controls (14/44 [32%] vs 22/25 [88%], *p* < 0.001) [35].

## Other Factors that May Impact the Immune Response to the COVID-19 Vaccine in the Context of Immune-Modifying Therapy

### Timing of Immune Response Readout Relative to Vaccination

The majority of studies in this review analysed immune responses to COVID-19 vaccines at 2 to 6 weeks following the first or second dose of the vaccine (Table 2). A smaller number of studies analysed immune responses at 7–8 days [19, 29] or up to 10 weeks following the second dose [31, 32].

### Immune Response to Different SARS-CoV-2 Variants

Of twenty-five studies included in this review, only four analysed the immune response against different SARS-CoV-2 variants [25, 26, 33•, 34•]. Dailey et al. (*n* = 33) found similar neutralising antibody responses following vaccination in an IBD population against wild-type and Alpha variants [26]. Our studies analysed neutralising activity against wild type, Alpha and Delta variants [33•, 34•]. Neutralising activity against wild-type SARS-CoV-2 was significantly lower after the first vaccine dose in patients receiving methotrexate compared to controls, but was preserved in those receiving targeted biologics. Neutralising titres against the Alpha variant were similarly low in all study participants [33•]. We subsequently found that neutralising antibody titres against wild-type, Alpha and Delta SARS-CoV-2 variants following the second vaccine dose were similar in patients receiving methotrexate, targeted biologics, and healthy controls [34•]. Chen et al. (individuals with IMIDs, *n* = 75; healthy controls, *n* = 25) also found no statistically significant differences in antibody responses in their IMID cohort against wild type, Alpha, Beta and Delta variants at 3 months following a second dose of vaccine [25]. Importantly, there remains a paucity of data on newer variants of concern. This remains an area of active investigation since the impact of a lower titre antibody vaccine response in the context of immune-modifying therapy is likely to be exacerbated when considering protection against newer variants such as Omicron [44].

### Omission of Immune-Modifying Therapy Around the Time of Vaccination

Prior studies found that temporary discontinuation of methotrexate for 2–4 weeks after seasonal influenza vaccination in patients with rheumatoid arthritis led to greater antibody titres [47, 48]. There has thus been active investigation into whether the omission of methotrexate doses around the time of COVID-19 vaccination is beneficial. The VROOM trial was a UK open-label two-arm parallel-group multicentre (*n* = 26 sites) randomised controlled superiority trial studying the effects on COVID-19 booster vaccine responses of temporarily suspending methotrexate for 2 weeks post-vaccination versus continued methotrexate [49•]. The study included 254 adults with IMIDs (127 in the ‘continue methotrexate’ group and 127 in the ‘suspend methotrexate’ group). Their mean age was 59 years, 155 (61%) were female, 130 (51%) had rheumatoid arthritis and 86 (34%) had psoriasis with or without arthritis. After 4 weeks, the mean S1-RBD antibody titre was 22,750 U/mL (95% CI 19,314–26,796) in the ‘suspend methotrexate’ group versus 10,798 U/mL (95% CI 8970–12 997) in the ‘continue methotrexate’ group (geometric mean ratio 2·19, 95% CI 1·57–3·04; *p* < 0·0001). Thus, a 2-week interruption of methotrexate treatment post-booster vaccine may lead to increased vaccine efficacy and greater duration of protection [49•]. A greater proportion of participants in the ‘suspend methotrexate’ group self-reported at least one disease flare in the first 4 weeks post-vaccination compared to the ‘continue methotrexate’ group (56% vs 31%); however, most flares were self-managed in both groups. Further research into the temporary suspension of other standard and targeted immune-modifying drugs around the time of vaccination is warranted.

### Total Number of Vaccine Doses

While there remains a paucity of data on the immune response to more than two COVID-19 vaccine doses, there are emerging reports. For example, Schmiedeberg et al. (*n* = 17) found that individuals with rheumatoid arthritis who were receiving standard systemic and/or targeted immune-modifying therapy who had not seroconverted after the first two COVID-19 vaccine doses mounted a significant antibody response following the third dose [50]. Wieske et al. showed that a third vaccine dose successfully boosted seroconversion rates in individuals receiving mycophenolate mofetil combination treatments, from 52.6% after the second vaccination to 89.5% after the third [41]. However, the study found that use of anti-CD20 biologics continued to impair humoral responses, with seroconversion rates of 36.9% and 45.6% after the second and third vaccine doses, respectively [41]. In further support, Simon et al. found that while a third vaccine enabled seroconversion in some individuals on rituximab who had not responded to previous vaccine doses, seroconversion rates remained lower in this group compared to previous non-responders who were not receiving rituximab (6/33 [18.2%] vs 26/33 [78.8%], respectively) [51].

With respect to clinical effectiveness, a study by Shen et al. using electronic health records in the Michigan Medicine US healthcare system (*n* = 154,519) found that individuals receiving immune-modifying drugs (standard systemic and/or targeted immune-modifying therapy) who had a third vaccine dose had a lower incidence of SARS-CoV-2 infection compared to those who did not have a third dose [52•]. Immune responses were not studied. Additionally, Risk et al.’s analysis of Michigan Medicine data (*n* = 168,414) indicated that three doses of BNT162b2 or mRNA-1273 vaccine was strongly protective against COVID-19-related hospitalisation in both individuals receiving immune-modifying therapy (vaccine effectiveness 87%, 95% CI 74–93) and those who were immunocompetent (92%, 95% CI 87–95) [53]. Further large-scale clinical effectiveness data from populations receiving immune-modifying therapies are awaited.

## Conclusions

SARS-CoV-2 has had a devastating effect around the world, with vaccinations being paramount in the public health strategy against COVID-19. While vaccination programmes have seen great success, there are still limited data on the clinical effectiveness of these vaccines in the global population who are receiving treatment with immune-modifying drugs, including those with skin IMIDs [54].

Research to date indicates that while individuals receiving immune-modifying therapy are more likely to have attenuated humoral responses to a single dose of COVID-19 vaccine compared to healthy controls, this may be improved by a complete course of vaccination. Hence, individuals receiving immune-modifying treatments should be encouraged to take up a complete vaccine course to mitigate their risk against severe COVID-19 outcomes [55]. Of specific note are B cell targeted biologics such as rituximab, which markedly impair the humoral response to both the first and second COVID-19 vaccination. Although this remains an area of active research, the impact of rituximab on vaccine responses may be improved by increasing the time interval between the rituximab treatment and vaccination [56]. Clinicians should also consider temporary cessation of methotrexate following vaccination given the emerging trial data [49•].

There remains a paucity of data on cellular immune responses, with the few available studies indicating lower responses to two vaccine doses in individuals receiving immune-modifying therapies compared to healthy controls, which may impact the durability of immune responses. Research in large patient populations on the longevity/kinetics of the complex humoral and cellular response to subsequent vaccine doses, including against newer variants of concern, is now warranted, in addition to data on immune correlates of vaccine clinical effectiveness.
